# Consolidation Chemotherapy Rather than Induction Chemotherapy Can Prolong the Survival Rate of Inoperable Esophageal Cancer Patients Who Received Concurrent Chemoradiotherapy

**DOI:** 10.3390/curroncol29090499

**Published:** 2022-09-02

**Authors:** Xiaojie Xia, Mengxing Wu, Qing Gao, Xinchen Sun, Xiaolin Ge

**Affiliations:** 1 Department of Radiation Oncology, The First Affiliated Hospital of Nanjing Medical University, 300 Guangzhou Road, Nanjing 210029, China; 2 The First School of Clinical Medicine, Nanjing Medical University, Nanjing 210029, China

**Keywords:** consolidation chemotherapy, induction chemotherapy, concurrent chemoradiotherapy, esophageal cancer

## Abstract

Concurrent chemoradiotherapy (CRT) is regarded as the standard treatment for inoperable esophageal cancers (EC). It is still controversial whether consolidation chemotherapy (CCT) or induction chemotherapy (IC) is beneficial for the patients who received CRT. Therefore, we carried out a retrospective analysis at our institution. A total of 186 inoperable EC patients from 20 October 2017 to 7 June 2021 who have previously received CRT were included in our study. The patients were divided into IC + CRT (n = 52), CCRT (n = 64), and CRT + CCT (n = 70) groups according to whether they received induction chemotherapy, consolidation chemotherapy, or not. We used Kaplan–Meier statistics to analyze their 1-, 2-, and 3-year OS. The median follow-up time for the whole group was 14.15 months. The 1-, 2-, 3- year overall survival (OS) for the CCRT group were 72.2%, 52.5%, and 29.5%, and 50.9%, 37.5%, and 25% for the IC + CRT group (*p* > 0.05). For the CRT + CCT group,1-, 2-, and 3-year OS were 89.8%, 59.0%, and 42.5% (*p* < 0.05). Adverse reactions in the three groups were mainly graded 0–3. The difference between the three groups was not statistically significant (*p* > 0.05). For non-surgical EC patients who received CRT, CCT after CRT but not IC before CRT can improve 1-, 2-, and 3-year OS with a low incidence of associated severe adverse effects. As a result, the addition of consolidation chemotherapy to chemoradiotherapy has significant prognostic advantages for inoperable EC patients.

## 1. Introduction

With increasing incidence and mortality, esophageal cancer (EC) has been the 8th most common tumor worldwide and the 2nd most common tumor of the digestive system after gastric cancer [1,2]. The main pathological types of EC are adenocarcinoma and squamous cell carcinoma. The former is mainly found in European and American countries, while the latter is mainly found in Asian countries such as China [3]. Early esophageal cancer is mainly treated by surgery. For operable locally advanced esophageal cancer, the CROSS study identified the standard modality of treatment for neoadjuvant chemoradiotherapy combined with surgery [4,5,6]. However, due to the lack of early clinical symptoms, many esophageal cancers are detected and diagnosed in advanced stages, at which time patients often have lost the opportunity for surgery. For these patients with locally advanced and inoperable esophageal cancer, concurrent chemoradiotherapy is considered to be the main treatment. The RTOG 85-01 trial reported a significantly higher 5-year survival rate for EC patients who received concurrent radiotherapy with cisplatin and fluorouracil than for those treated with radiotherapy alone(26% vs. 0%) [7]. In spite of this, the locoregional recurrence rate (LRR) of EC patients receiving concurrent chemoradiotherapy (CRT) is as high as 40–60% [8] and the 5-year survival rate is only about 10–30% [9]. Therefore, induction chemotherapy (IC) and consolidation chemotherapy (CCT) have been used in combination with concurrent chemoradiotherapy to improve survival outcomes for patients with esophageal cancer.

With that in mind, what effects do induction chemotherapy and consolidation chemotherapy have on patients with esophageal cancer with CRT? Theoretically, IC is thought to improve dysphagia, reduce tumor volume, eliminate micrometastasis, test the effect of chemotherapy, and create survival benefits [10,11]. Some studies have shown that patients treated with IC + CRT, especially IC responders, had significantly higher overall survival (OS) and progression-free-survival (PFS) than those in the CRT group, showing a superior survival advantage [12,13]. However, other studies have reported that IC did not significantly improve OS, local failure-free survival (LFS), and distant failure-free survival (DFS) [14], which may even reduce the dose intensity of CRT and increase postoperative morbidity and mortality [15]. Therefore, the clinical efficacy of induction chemotherapy is still controversial. CCT refers to the use of additional drugs at the end of a prescribed initial therapy cycle to prolong the duration of chemotherapy after patients have reached the maximum tumor response [16]. With the efficacy of CCT in cervical cancer [17] and lung cancer gradually emerging [18,19], the role of CCT in the treatment of esophageal cancer is also being revealed. Two meta-analyses published in 2021 also demonstrated that patients with esophageal cancer can achieve survival benefits in CCT after CRT [20,21].

Whether it is induction chemotherapy or consolidation chemotherapy, there are not many clinical studies related to esophageal cancer, and there is a lack of relatively large or prospective randomized controlled trials, so their adjuvant effects on simultaneous chemoradiotherapy are not very clear. Therefore, we retrospectively analyzed the survival rates and toxic side effects of patients with esophageal squamous cell carcinoma (ESCC) receiving IC + CRT, CRT alone, and CRT + CCT to further determine the clinical efficacy of IC and CCT in ESCC patients who received CRT.

## 2. Materials and Methods

### 2.1. Pretreatment Staging

Tumors were clinically staged by endoscope and ultrasonography, barium esophagography, and enhanced computed tomography (CT) as per the 8th edition of the Union for International Cancer Control TNM. Only 12 patients had PET-CT in their pretreatment staging and the others were unable to have this test due to financial constraints. Tumor locations were separated into the cervical, upper, middle, and lower esophagus.

### 2.2. Chemotherapy

All patients received current chemotherapy. Experienced clinicians decided the single- or double-drug chemotherapy regimen according to each patient’s condition. The single- and double-drug regimens mainly include S-1 or taxane; taxane plus cisplatin, raltitrexed plus cisplatin, or S-1 plus cisplatin. Both paclitaxel and docetaxel were used in our concurrent chemotherapy regimen. In our study, the most used drug was cisplatin. Clinicians chose the appropriate regimen according to the state of illness, economic status, and physical condition. Each cycle lasted three weeks, and two cycles were separated by two weeks. Some patients underwent induction chemotherapy (IC) and adjuvant chemotherapy (ACT). Clinicians decreased the dose or stopped the scheme when necessary. In case of relapse after concurrent CRT, consolidation chemotherapy was performed, and the specific plan was determined by the experienced clinicians.

### 2.3. Radiation Therapy

All patients underwent IMRT offered by a 6-MV linear accelerator. Gross tumor volume (GTV) referred to the macroscopic original tumor and local lymph node metastases. The clinical target volume (CTV) was derived from GTV by prolonging the radiating coverage by 1 cm laterally and 5 cm both inferiorly and superiorly. A 1 cm margin was set around the pathological lymph nodes. Planning target volume (PTV) referred to CTV plus errors of tumor shift and placement due to organ spontaneous and involuntary motions. PTV was defined by prolonging the CTV by about 0.3 cm radially and 0.5 cm both distally and proximally. The treatment plan and dose limits of organs at risk were based on the National Comprehensive Cancer Network version 1, 2020. Tumors were evaluated with barium esophagography and enhanced CT when the treatment dose of the lesion reached 50 Gy. If there were residual lesions, the total dose was increased appropriately. For patients with large lesions and high doses to the organs at risk, the treatment dose was lowered appropriately to reduce the possibility of radiotherapy-related side effects. The median radiation dose offered in 5 weeks was 60 Gy.

### 2.4. Endpoints and Follow-Up

The primary endpoints were OS (from the date of treatment to the date of death or last contact). Efficacy was assessed every 6 months up to August 2021 or until death.

### 2.5. Statistics

Statistical analyses were finished on SPSS 26 (IBM Corp., Armonk, NY, USA). We compared the differences in each characteristic between the two groups by chi-square test. OS was computed by the Kaplan–Meier approach and examined by a log-rank test. Significance was set at two-sided *p* < 0.05 (95% confidence interval CI).

## 3. Results

### 3.1. Patients and Tumor Characteristics

A total of 186 inoperable EC patients (52 in the IC + CRT group, 64 in the CCRT group, and 70 in the CRT + CCT group) were treated between October 2017 and June 2021. Details are shown in Table 1. We compared the three groups by gender, age, smoking and alcohol status, ECOG score, tumor diameter, tumor location, T-, N-staging, clinical stage, and Radiation dose. The baseline of patients and tumor characteristics in the three groups were well balanced (*p* > 0.05). The median age was 76 years (range 53–88 years) in the CCRT group, 66 (range 49–80 years) in the IC + CRT group, and 65 (range 55–85 years) in the CRT + CCT group. As shown in Table 1, most patients were diagnosed in the late stage and radiotherapy doses were usually ≤60 Gy.

### 3.2. Survival Outcomes

**Figure 1:**
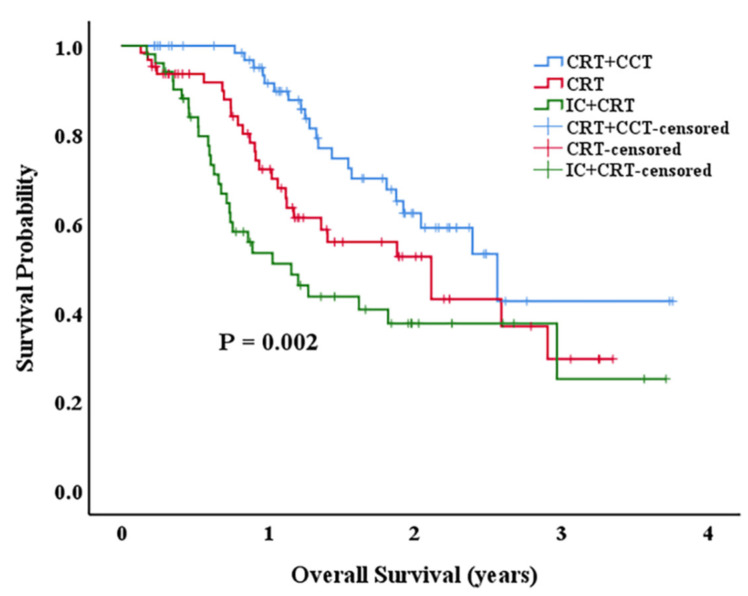
OS of patients for IC + CRT, CRT, and CRT + CCT group.

The 1-, 2-, and 3-year OS rates were higher in the CRT + CCT group compared to the CCRT and IC + CRT group (Figure 1) (*p* = 0.002). The 1-year, 2-year, and 3-year OS for the CCRT group were 72.2%, 52.5%, and 29.5%, and 50.9%, 37.5%, and 25% for the IC + CRT group (*p* > 0.05). For the CRT + CCT group, 1-year, 2-year, 3-year OS were 89.8%, 59.0%, and 42.5% (*p* < 0.05), respectively.

### 3.3. Toxicity of Treatment

The hematological and nonhematological toxicities are presented in Table 2. The hematological toxicities mainly include leukopenia, thrombocytopenia, anemia, and the increase of alanine aminotransferase (ALT) or aspartate aminotransferase (AST). Nausea, vomiting, radiation esophagitis, radiation pneumonia, and so on belong to nonhematological toxicities. As we can see, the severe toxic effects (grade 4) in the three groups were seldom raised. One case of grade 4 ALT/AST increased and vomiting occurred in the IC + CRT group and one case of grade 4 nausea condition occurred in the CCRT group, while grade 4 leukopenia, nausea, and radiation esophagitis occurred in the CRT + CCT group. There were no significant differences in the occurrence of hematological and nonhematological toxicities between the two groups (all *p* > 0.05).

## 4. Discussion

By 2020, there were 604,100 new cases of esophageal cancer worldwide, accounting for 3.1% of all new cancers, and 54,076 new deaths, accounting for 5.5% of all new deaths [2]. In addition to traditional surgery, radiotherapy, and chemotherapy, immunotherapy and targeted therapy have gradually been used in the clinical treatment of esophageal cancer, but concurrent radiotherapy and chemotherapy is still the standard treatment for locally advanced inoperable esophageal cancer. There is still no unified conclusion about the effect of induction chemotherapy and consolidation chemotherapy combined with concurrent chemoradiotherapy. To the best of our knowledge, this is the first clinical study that reports the outcomes and safety of IC + CRT, CRT, and CRT + CCT in patients with patients with locally advanced inoperable ESCC.

A retrospective study conducted by Luo et al. found that the median OS (26.0 vs 22.0 months) and 3-year OS (30.6% vs. 25.9%) of patients treated with IC + CCRT were significantly higher than those of the CCRT group [12]. Our study provides the opposite conclusion: the IC + CRT group receiving paclitaxel or raltitrexed in combination with platinum did not have a significant improvement in OS (*p* = 0.114). This is similar to what some reports have found [14,22,23]. The reasons for this divergence may be that: (1) The inconsistency of chemotherapy regimens leads to differences in the intensity and efficacy of treatment; (2) The sample size is too small so that the efficacy of the IC cannot be fully reflected; (3) Selection of population. Patients with late-stage are more likely to benefit from IC [24]; (4) Differences in pathologic types. Patients with adenocarcinoma seem to be more likely to benefit from IC [25,26]. A randomized Phase III prospective study currently under recruitment in Japan aims to compare patients undergoing transit surgery or CRT after docetaxel plus cisplatin and 5-fluorouracil (DCF) induction chemotherapy with patients with CRT alone to demonstrate that the overall survival (OS) of induced chemotherapy is superior to CRT alone [27]. We expect this study to provide new evidence for the clinical efficacy of IC.

Although to our findings, IC-CRT does not improve OS of patients with esophageal squamous cell carcinoma, treatment options for CCT after CRT shows good efficacy. The 1-year, 2-year, and 3-year survival rates of the CRT group and the CRT + CCT group were 72.2% and 89.8%, 52.5% and 59.0%, 29.5% and 42.5%, respectively, and the OS of the two groups were significantly different (*p* = 0.04). Relevant retrospective clinical studies reported consistent findings [28,29,30]. A recent meta-analysis of 11 articles shows that although CCT did not improve the disease control rate (DCR) (*p* = 0.384) and the objective response rate (ORR) (*p* = 0.393), it significantly improved OS (*p* < 0.001) and PFS (*p* = 0.003) [20]. The DCR and ORR were derived from 368 patients in 3 studies, whereas OS and PFS were respectively obtained from 2008 patients in 11 studies and 1111 patients in 6 studies. We consider the results of the meta-analysis of DCR and ORR to be doubtful because of the small sample size and heterogeneity. The positive results of OS and PFS may be due to the fact that CCT further removed tumor cells from the blood and reduced distant metastases. Although CCT did not significantly improve OS in some trials, the OS of the CRT + CCT group was still prolonged compared with the CRT group alone, indicating that CCT slowed down the development of tumors to some degree, and had the potential to prolong the survival of patients [31]. Hopefully, a prospective, randomized, controlled phase III trial comparing CRT plus CCT with CRT alone for locally advanced esophageal cancer is currently underway and we look forward to its results [32]. In our study, the CRT + CCT group only underwent two cycles of consolidation chemotherapy. However, in addition to confirming the effect of CCT, the study of Zhang AD et al. also found that the survival benefit of patients receiving three to four cycles of CCT was more remarkable (*p* = 0.011), and they recommended completing no less than two cycles of consolidation chemotherapy if possible [30]. Nevertheless, in the actual case, the optimal number of cycles of consolidation chemotherapy is still to be discussed because the patient’s physical condition, economic status, and other relevant factors need to be considered. Additionally, a study published in 2022 compared the efficacy and safety of IC + CRT, IC + CRT + CCT, and CRT + CCT for the first time, suggesting that IC + CRT + CCT may also be a new treatment mode that can be tried [33].

In terms of side effects, our study provided similar results to previous reports [12,34,35]. The adverse events of the selected patients were mainly manifested as myelosuppression, nausea, vomiting, radiation esophagitis, and radiation pneumonia. There were no significant differences in the incidence of side effects among the three groups and they were all controllable. This result showed that the treatment of IC + CRT, CRT, and CRT + CCT was safe and feasible.

This study had several limitations. First, the study is retrospective and it was conducted in a single institution. Second, the sample size is not very large. Third, induction and consolidation chemotherapy were performed in two cycles in this paper so the treatment of other cycles was not investigated.

## 5. Conclusions

Our research proves that for patients with inoperable esophageal squamous cell carcinoma, two cycles of IC before CRT does not prolong their survival, while two cycles of CCT after CRT can improve OS significantly and safely. Our conclusion requires support from further large prospective studies.

## Figures and Tables

**Table 1:** Basic characteristics of IC + CRT, CRT, and CRT + CCT groups.

Project	Patient (Range, %)						*P*
	IC + CRT (n = 52)		CRT (n = 64)		CRT + CCT (n = 70)		
Age							0.41
Median	66 (49–80)		76 (53–88)		65 (55–85)		
<60	9	17.31%	6	9.38%	11	15.71%	
≥60	43	82.69%	58	90.63%	59	84.29%	
Gender							0.472
Female	16	30.77%	18	28.13%	15	21.43%	
Male	36	69.23%	46	71.88%	55	78.57%	
Smoking							0.97
Yes	28	53.85%	33	51.56%	37	52.86%	
No	24	46.15%	31	48.44%	33	47.14%	
Alcohol status							0.942
Yes	26	50.00%	31	48.44%	36	51.43%	
No	26	50.00%	33	51.56%	34	48.57%	
ECOG							0.386
0	18	34.62%	19	29.69%	24	34.29%	
1	32	61.54%	38	59.38%	36	51.43%	
2	2	3.85%	7	10.94%	10	14.29%	
Tumor diameter							0.661
<5cm	12	23.08%	16	25.00%	21	30.00%	
≥5cm	40	76.92%	48	75.00%	49	70.00%	
Location							0.098
Cervical	1	1.92%	3	4.69%	3	4.29%	
Upper	14	26.92%	15	23.44%	32	45.71%	
Middle	15	28.85%	15	23.44%	14	20.00%	
Lower	21	40.38%	28	43.75%	21	30.00%	
Two pieces	1	1.92%	3	4.69%	0	0.00%	
T-staging							0.156
T1	0	0.00%	1	1.56%	1	1.43%	
T2	5	9.62%	3	4.69%	13	18.57%	
T3	21	40.38%	32	50.00%	30	42.86%	
T4	26	50.00%	28	43.75%	26	37.14%	
N-staging							0.609
N0	19	36.54%	33	51.56%	32	45.71%	
N1	24	46.15%	23	35.94%	26	37.14%	
N2	8	15.38%	5	7.81%	9	12.86%	
N3	1	1.92%	3	4.69%	3	4.29%	
Clinical staging							0.554
Ⅰ	0	0.00%	1	1.56%	1	1.43%	
II	13	25.00%	22	34.38%	26	37.14%	
III	13	25.00%	11	17.19%	17	24.29%	
IVA	26	50.00%	30	46.88%	26	37.14%	
Radiation dose							0.074
≤60 Gy	49	94.23%	64	100.00%	65	92.86%	
>60 Gy	3	5.77%	0	0.00%	5	7.14%	

IC, Induction chemotherapy; CRT, Concurrent chemoradiotherapy; CCT, Consolidation chemotherapy.

**Table 2:** Toxicity of patients for IC + CRT, CRT, and CRT + CCT groups.

Toxicity	IC + CRT (n = 52)				CRT (n = 64)				CRT + CCT (n = 70)				*p* Value
	Grade 0–1	Grade 2	Grade 3	Grade 4	Grade 0–1	Grade 2	Grade 3	Grade 4	Grade 0–1	Grade 2	Grade 3	Grade 4	
leukopenia	26	12	12	0	23	9	9	0	34	13	14	1	0.997
thrombocytopenia	28	8	14	0	24	9	8	0	36	14	12	0	0.769
anemia	28	22	0	0	24	16	1	0	36	24	1	0	0.913
ALT/AST	6	2	1	1	9	2	1	0	10	3	2	0	0.957
nausea	37	9	4	0	28	6	6	1	17	10	9	1	0.091
vomiting	23	20	2	1	22	18	1	0	25	27	2	0	0.936
radiation esophagitis	27	23	1	0	23	16	1	0	28	25	1	1	0.98
radiation pneumonia	34	5	3	0	32	3	3	0	49	6	5	0	0.995

ALT, Alanine aminotransferase; AST, Aspartate aminotransferase.

## Data Availability

The original contributions presented in the study are included in the article. Further inquiries can be directed to the corresponding authors.
